# Soccer athletes are superior to non-athletes at perceiving soccer-specific and non-sport specific human biological motion

**DOI:** 10.3389/fpsyg.2015.01343

**Published:** 2015-09-03

**Authors:** Thomas Romeas, Jocelyn Faubert

**Affiliations:** Visual Psychophysics and Perception Laboratory, School of Optometry, Université de Montréal, MontrealQC, Canada

**Keywords:** perceptual-cognitive expertise, sport performance, point-light walker, point-light soccer, discrimination task, skill transfer

## Abstract

Recent studies have shown that athletes’ domain specific perceptual-cognitive expertise can transfer to everyday tasks. Here we assessed the perceptual-cognitive expertise of athletes and non-athletes using sport specific and non-sport specific biological motion perception (BMP) tasks. Using a virtual environment, university-level soccer players and university students’ non-athletes were asked to perceive the direction of a point-light walker and to predict the trajectory of a masked-ball during a point-light soccer kick. Angles of presentation were varied for orientation (upright, inverted) and distance (2 m, 4 m, 16 m). Accuracy and reaction time were measured to assess observers’ performance. The results highlighted athletes’ superior ability compared to non-athletes to accurately predict the trajectory of a masked soccer ball presented at 2 m (reaction time), 4 m (accuracy and reaction time), and 16 m (accuracy) of distance. More interestingly, experts also displayed greater performance compared to non-athletes throughout the more fundamental and general point-light walker direction task presented at 2 m (reaction time), 4 m (accuracy and reaction time), and 16 m (reaction time) of distance. In addition, athletes showed a better performance throughout inverted conditions in the walker (reaction time) and soccer kick (accuracy and reaction time) tasks. This implies that during human BMP, athletes demonstrate an advantage for recognizing body kinematics that goes beyond sport specific actions.

## Introduction

In sport, expertise is defined as consistent superior athletic performance over an extended period (Wells et al., 2014). Sport science has demonstrated that some individuals can develop special expertise due to extensive experience to highly specific action patterns (Sparrow and Sherman, 2001). In this regard, elite athletes can be considered as a striking example of higher expertise in specific action recognition.

One of the most remarkable capacities of experts in sports is their ability to quickly and accurately determine the key characteristics of motion which is a fundamental property of the visual system (Dittrich, 1993; Sparrow and Sherman, 2001). A number of studies has reported that elite athletes possess superior perceptual-cognitive skills compared to sub-elite and/or novices in sports-specific tasks including advance visual cue utilization (Williams, 2000; Abernethy et al., 2001; Ward et al., 2002), pattern recall and recognition (Smeeton et al., 2004; Abernethy et al., 2005), visual search strategies (Williams, 2000; Vaeyens et al., 2007) and the knowledge of situational probabilities (Williams et al., 2006; North and Williams, 2008). Moreover, this perceptual-cognitive expertise has also been evoked and transferred in a sport-free context since athletes outperformed non-athletes in socially realistic multitasking crowd scenes involving pedestrians crossing streets (Chaddock et al., 2011) or in learning complex and neutral dynamic visual scenes (Faubert, 2013). Overall, perceptual-cognitive expertise has been widely reported using specific or context-free paradigms in different kinds of sports (e.g., Starkes, 1987; Helsen and Starkes, 1999; Williams et al., 1999; Ward and Williams, 2003; Mann et al., 2007; Voss et al., 2010; Alves et al., 2013; Romeas et al., 2015).

A number of studies have highlighted the perceptual-cognitive expertise of athletes in sport-specific context by using biological motion perception (BMP) tasks (e.g., Abernethy et al., 2001; Ward et al., 2002; Abernethy and Zawi, 2007; Calvo-Merino et al., 2010; Hohmann et al., 2011). The term BMP was first introduced by Johansson (1973) in an attempt to characterize the movement patterns obtained from humans or more generally from animate beings (Johansson, 1973). BMP involves the visual systems’ capacity to recognize the kinematic presentation of the human or animal movements reduced to a few moving dots placed on the major joints of the body (Bellefeuille and Faubert, 1998; Ptito et al., 2003). When in motion, isolated points of light on the joint centers give a compelling impression of the action. This representation allows human observers to recognize complex actions spontaneously from various animations such as a walking human. BMP enables us to determine what the observer perceives solely on kinematics, while other motion cues are eliminated (Sparrow and Sherman, 2001) and it was shown to be equally effective as when the full body contours are present during action (Bellefeuille and Faubert, 1998) or as when the information is displayed through a video presentation (Munzert et al., 2010). This task is recognized as a critical and fundamental ability of social relevance (Troje, 2002), and is a very strong dynamic cue that has been used, among others (for a review see Troje, 2013), for collision avoidance (Ouellette et al., 2009) or to highlight athlete expertise in sport science. Furthermore, biological motion studies have shown potential for the study of spatial characteristics of perception related to sport action (Abernethy and Parker, 1989; Ward et al., 2002; Wright et al., 2011) and has allowed researchers to assess perception of sport action within a life-sized virtual environment using stereoscopic displays (Bideau et al., 2010; Ida, 2012). The use of virtual stimuli gives the participant vital depth information and corresponds more closely to the players’ perspective and behavior in real-life environments. For instance, it has been shown that walker dimensions corresponding to a different person at different distances from the observer varying from 1 to 16 m can generate dramatic performance differences in some populations (Legault and Faubert, 2012; Legault et al., 2012). In sports, Bideau et al. (2003) showed that an interactive, immersive virtual handball court with a realistically animated handball player (from motion capture) throwing the ball toward the goal elicited expert handball goalkeeper responses similar to real-world responses. Virtual reality involves stereoscopy (binocular disparity) which is required in situations where fast, complex and dynamic elements overlap (e.g., body joints in motion). For instance, stereoscopy has been shown to help disambiguate object occlusions when processing abstract dynamic visual scenes (Faubert and Allard, 2013). It is also suggested that stereoscopic information critically affects human perceptual motor performance. For instance, it has been shown that good stereo vision allows for significant learning enhancement during a tennis ball catching task when comparing to individuals with poor stereo acuity (Mazyn et al., 2007). Stereoscopy gives explicit depth cues that can help to disambiguate the perception of biological motion and avoid ambiguity (facing toward the viewer bias) in facing experiments (Vanrie et al., 2004; Jackson and Blake, 2010; de Lussanet and Lappe, 2012). To our knowledge, no study has yet to explore athlete’s expertise in both sport-specific and non-sport specific BMP contexts using virtual reality.

In the present study, we aimed to assess the degree of expertise between soccer players and non-athletic young adults in a virtual environment with two biological motion facing tasks slightly different in nature: a point-light walker and a point-light soccer kick. Whereas one stimulus represents an everyday task, e.g., perception of a walking human, the other one belongs to a specific action pattern category that is thought to require some expertise for efficient processing. Evidence suggests that BMP should be influenced by the observers’ familiarity with the recognized action (Calvo-Merino et al., 2010). Moreover, it is known that the human perceptual system can learn a very subtle BMP task, based solely on the previous visual experience, but also even more strongly on motor experience (Calvo-Merino et al., 2010). Here, we are interested to see if athletes’ perceptual-cognitive expertise can also benefit to non-sport specific context as it was recently suggested that they are better at perceiving human body movements (Wei et al., 2011). Based on previous evidence, we hypothesized that athletes would perform better than non-athletes in predicting the trajectory of a masked soccer ball based solely on the body kinematics of the kicker (sport-specific expertise). In addition, we think that athletes’ perceptual-cognitive expertise could expand to a greater general context such as the perception of a normal human walker’s kinematics which is void of sport related action. Young soccer players and non-athlete adults were tested using the aforementioned biological motion facing tasks in which the angle and distance of presentation were varied in order to modify task difficulty (see Saunders et al., 2010; Legault et al., 2012).

## Materials and Methods

### Participants

Fifty-nine adults participated in the study, including forty university soccer players and nineteen non-athletes. All subjects reported normal or corrected-to-normal vision (6/6 or better) with normal stereoacuity (50 s of arc or better). Participant levels of physical activity are reported in **Table 1**. None of the subjects had previous experience with biological motion displays. The experimental protocol and related ethics issues were evaluated and approved by the Comité d’Éthique de la Recherche en Santé of Université de Montréal and were carried out in accordance with the World Medical Association Helsinki Declaration. All subjects were given verbal and written information on the study and gave their verbal and written informed consent to participate.

**Table 1:** Participants’ information (± SEM).

Participant	*n*	Mean Age (years)	Hours of weekly physical training
Athletes	40	21.51 ± 0.32	9.03 ± 0.69
Non-athletes	19	24.21 ± 0.50	1.42 ± 0.41

### Apparatus

The biological motion task was conducted using a fully immersive virtual environment (EON Icube^TM^). The EON Icube^TM^ is a 7 × 10 × 10 feet room that includes three rigid back projection surface walls (one frontal and two laterals) and a reflective floor. Four high-resolution projectors were synchronized and the image was updated in real-time to maintain the true viewing perspective of the observer. The EON Icube^TM^ was under the computer control of an Intel Xeon E5530 (NVIDIA Quadro FX 5800 graphic card) along with four Hewlett Packard Z800 workstations generating a stereoscopic environment. The stereoscopy was generated with CrystalEyes^®^ 4 s (RealD) active shutter glasses synchronized at 120 Hz.

### Stimuli

**FIGURE 1:**
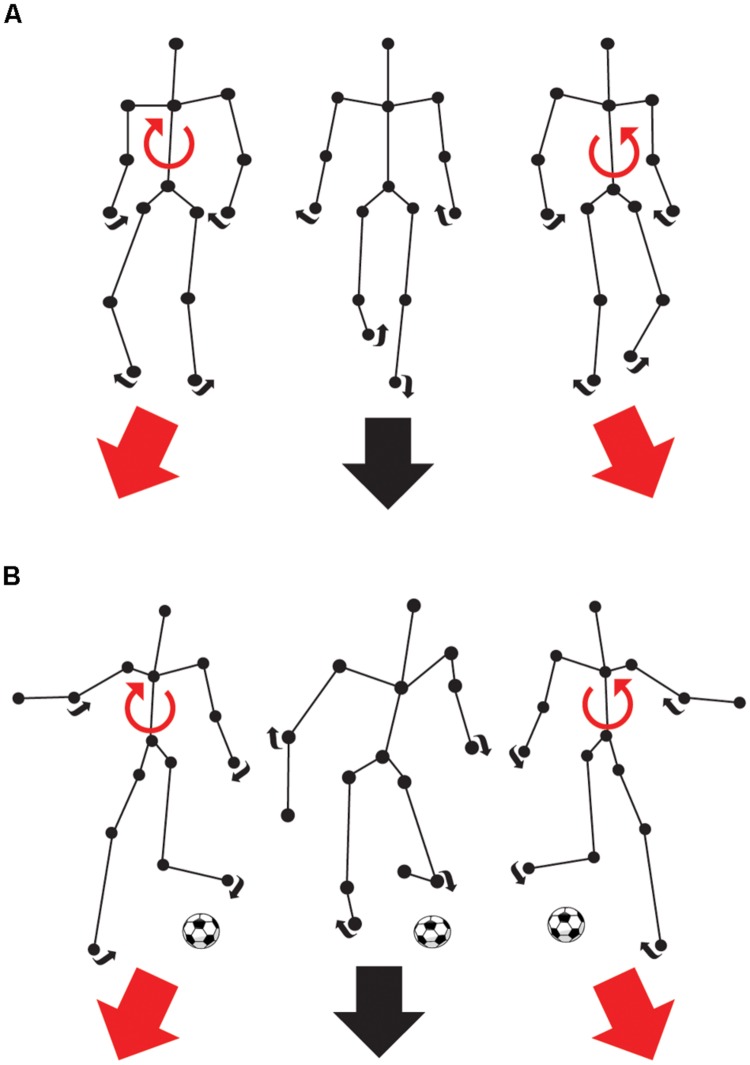
**Front-facing biological motion perception (BMP) tasks.** The connecting black lines and the balls are used here as a visual aid and were not presented during the experiment. **(A)** The task consists of choosing whether an animated point-light walker is walking rightward or leftward from the subjects own vertical reference. In short, the task is to determine whether the walker’s predicted path will end up to the left or the right of the subject’s own vertical center of reference. **(B)** The task consists of choosing whether an animated point-light soccer player is kicking a ball to the right or to the left of the subject’s own vertical center of reference.

The biological motion front-facing task (**Figure 1**) consisted of the discrimination of the direction (right or left) of a point-light walker and a point-light soccer kick. The point-light walker (Troje, 2008; Legault et al., 2013) and soccer kick (adapted from https://www.mixamo.com/ motion capture studio) were dynamic representations of human forms and were made up of 15 black dots, which represented the head, shoulders, hips, elbows, wrists, knees, and ankles on a white background. Each dot had a diameter of 0.1 m. The height of the point-light walker and soccer kick was 1.80 m disposed at a virtual distance from the observer of 2, 4, and 16 m subtending 42, 24, and 6.4° of visual angle, respectively. The duration of the presentation lasted for 1 s and contained 30 (walker) or 46 (soccer) frames. In the soccer task, the foot-to-ball contact moment was provided at 0.6 s. The inter-stimulus interval was 500 ms. Point-light walkers and soccer kicks were, respectively, presented walking or kicking leftward or rightward (forced choice paradigm). A constant stimuli procedure with random angles of presentation across trials was used for the point-light walkers (-6, -4, -2, 0, 2, 4, and 6° from front-facing) and the point-light soccer kicks (-15, -8, -4, -2, 0, 2, 4, 8, and 15° from front-facing). All of the angles were randomly presented forty times in each experimental block and their order of presentation varied according to the constant stimuli procedure. In each block, the distance and orientation was held constant. For each one of the two BMP tasks, there were six blocks in total that were classified according to distance (2, 4, and 16 m) and orientation (upright and inverted) such as 2 m upright, 4 m upright, 16 m upright, 2 m inverted, 4 m inverted, 16 m inverted.

### Procedure

Two sessions were used to separately evaluate the point-light walker and the point-light soccer kick tasks. One to seven days were allowed between each session. The order of session was randomized between subjects. During one session, each observer randomly started with either the blocks for the three upright distance conditions followed by the three inverted distance conditions or the opposite. The presentation of the blocks for the three distances was also randomized for each participant. A session lasted from about 45 min (walker) to 1 h (soccer) including small breaks (1 min) between each one of the six blocks of presentation. Each participant sat at 1.2 m from the EON Icube^TM^’s central wall with eye height at 1.45 m from the ground. They were asked to wear stereoscopic goggles and to fixate straight ahead. One practice block including 10 trials was then presented before each session, in which the participants had to efficiently and quickly identify between the direction of the point-light walker or the direction of the point-light soccer kick. As in the practice trial, each observer’s task consisted of a forced choice paradigm by discriminating the point-light walker (walking leftward or rightward relative to the observer) and soccer kick’s (kicking leftward or rightward relative to the observer) direction as efficiently and as quickly as possible, using an Xbox 360 controller including a left hand-button (left bumper for left answers) and a right hand-button (right bumper for right answers).

### Analysis

#### Response Accuracy

##### Procedure

Percentages of response accuracy for point-light walker and soccer kick were averaged across negative and positive corresponding angles because they were proportionally distributed (no bias was observed). For each participant and each condition, we plotted a fit using a non-linear regression (logistic function) with the software CurveExpert Professional 2.2.0. We then extrapolated the value corresponding to the just-noticeable difference (75% of response accuracy) in addition to the corresponding slope and used them as dependant variables (angular threshold and slope) to compare the two groups. We also extrapolated the value of response accuracy corresponding to the maximal angle of presentation (6° for walker, 15° for soccer kick) to compare upright and inverted condition because, in some inverted conditions, the just-noticeable difference was not reached. We performed the analysis using IBM SPSS statistics v19. We used parametric tests when the homogeneity of variances (Levene’s test) was non-significant. Otherwise, we used non-parametric tests.

##### Walker

A mixed-design analysis of variance (ANOVA) with repeated measures with the between-subject factor group (Athletes and Non-athletes) and the within-subject factor distances (2, 4, and 16 m) was applied on the angular threshold (for 75% of response accuracy). This was to compare the accuracy of performance between groups in the upright condition. Differences between groups were determined using independent *t*-tests for each distance of presentation. We used non-parametric Mann–Whitney *U*-tests on the slope values to compare differences between groups.

##### Soccer kick

We used non-parametric Mann–Whitney *U*-tests on the angular threshold values (for 75% of response accuracy) to compare differences between groups for each distance of presentation in the upright condition. A mixed-design ANOVA with repeated measures with the between-subject factor group (Athletes and Non-athletes) and the within-subject factor distances (2, 4, and 16 m) was applied on the slopes. We used independent *t*-tests to determine differences between groups. Moreover, three non-athletes participants were not taken into account throughout this analysis because their score did not reach the just-noticeable difference due to the difficulty of the task.

##### Walker vs. soccer kick

A repeated measures ANOVA with the within-subject factors tasks (walker, soccer kick) and distances (2, 4, and 16 m) was applied on the angular threshold (for 75% of response accuracy) and slope.

##### Inversion effect

A mixed-design ANOVA with repeated measures with the between-subject factor group (Athletes and Non-athletes) and the within-subject factor distances (2, 4, and 16 m) and orientations (upright, inverted) was applied on response accuracy (for maximal angle) for the walker task to mainly confirm the inversion effect. Differences between groups were determined using independent *t*-tests for each distance of presentation in the inverted condition. To assess the inversion effect in the soccer kick task, a non-parametric Wilcoxon test was used to compare the response accuracy (for maximal angle) between upright and inverted conditions for each distance of presentation. Differences in the inverted condition between groups were determined using a Mann–Whitney *U*-test for each distance of presentation.

#### Reaction time

##### Procedure

Reaction time of each participant was averaged for distance (2, 4, 16 m) and orientation (upright, inverted) conditions. To perform the analysis, we used parametric tests when the homogeneity of variances was non-significant (Levene’s test). Otherwise, we used non-parametric tests.

##### Walker

We used a non-parametric Mann–Whitney *U*-test to compare differences in reaction time between groups for each distance of presentation in the upright condition.

##### Soccer kick

A mixed-design ANOVA with repeated measures with the between-subject factor group (Athletes and Non-athletes) and the within-subject factor distances (2, 4, and 16 m) was applied on the reaction time. Differences between the two groups were determined using independent *t*-tests for each distance of presentation in the upright condition.

##### Walker vs. soccer kick

A repeated measures ANOVA with the within-subject factors tasks (walker, soccer kick) and distances (2, 4, and 16 m) was applied on the reaction time.

##### Inversion effect

To assess the inversion effect in the walker task, a non-parametric Wilcoxon test was used. Differences in the inverted condition between groups were determined using a Mann–Whitney *U*-test for each distance of presentation. For the soccer kick task, a mixed-design ANOVA with repeated measures with the between-subject factor group (Athletes and Non-athletes) and the within-subject factor distances (2, 4, and 16 m) and orientations (upright, inverted) was applied, mainly to confirm the inversion effect. Differences between groups in the inverted condition were determined using independent *t*-tests for each distance of presentation.

## Results

### Response Accuracy (**Table 2**)

**Table 2:** Mean (± SEM) response accuracy (angular threshold [75%], slope and response accuracy for maximal angle) between groups in the two tasks.

Response accuracy							

Point-light		Walker			Soccer kick		
**Group**		**Athletes**	**Non-athletes**	***p-*value**	**Athletes**	**Non-athletes**	***p-*value**
Distance and orientation	∙ Angular threshold (just-noticeable difference in °) for 75% of response accuracy					
	∙ Slope					
	
	2 m Upright	2.25 ± 0.16	2.86 ± 0.32	~^∗^0.058	10.08 ± 0.87	21.30 ± 4.80	0.108
		10.47 ± 0.84	8.61 ± 1.18	0.194	3.43 ± 0.36	2.35 ± 0.43	0.093
	
	4 m Upright	1.69 ± 0.14	2.24 ± 0.25	^∗^0.045	9.64 ± 0.94	20.21 ± 4.76	^∗^0.048
		14.44 ± 1.20	10.10 ± 1.24	^∗^0.046	3.30 ± 0.27	2.31 ± 0.30	^∗^0.042
	
	16 m Upright	1.76 ± 0.13	1.69 ± 0.16	0.783	8.01 ± 0.62	18.08 ± 3.46	^∗^0.009
		12.75 ± 1.03	10.94 ± 1.63	0.236	4.23 ± 0.41	2.28 ± 0.39	^∗^0.006
	
	∙ Response accuracy (%) at	6°			15°		
	
	2 m Inverted	77.62 ± 2.51	70.83 ± 4.45	0.151	71.85 ± 2.66	60.21 ± 2.49	^∗^0.017
	4 m Inverted	76.36 ± 2.65	68.57 ± 4.69	0.119	71.74 ± 2.55	60.70 ± 3.18	^∗^0.012
	16 m Inverted	63.89 ± 2.51	60.18 ± 2.98	0.372	72.14 ± 2.72	61.95 ± 2.85	^∗^0.031

*p < 0.05.

#### Walker

**FIGURE 2:**
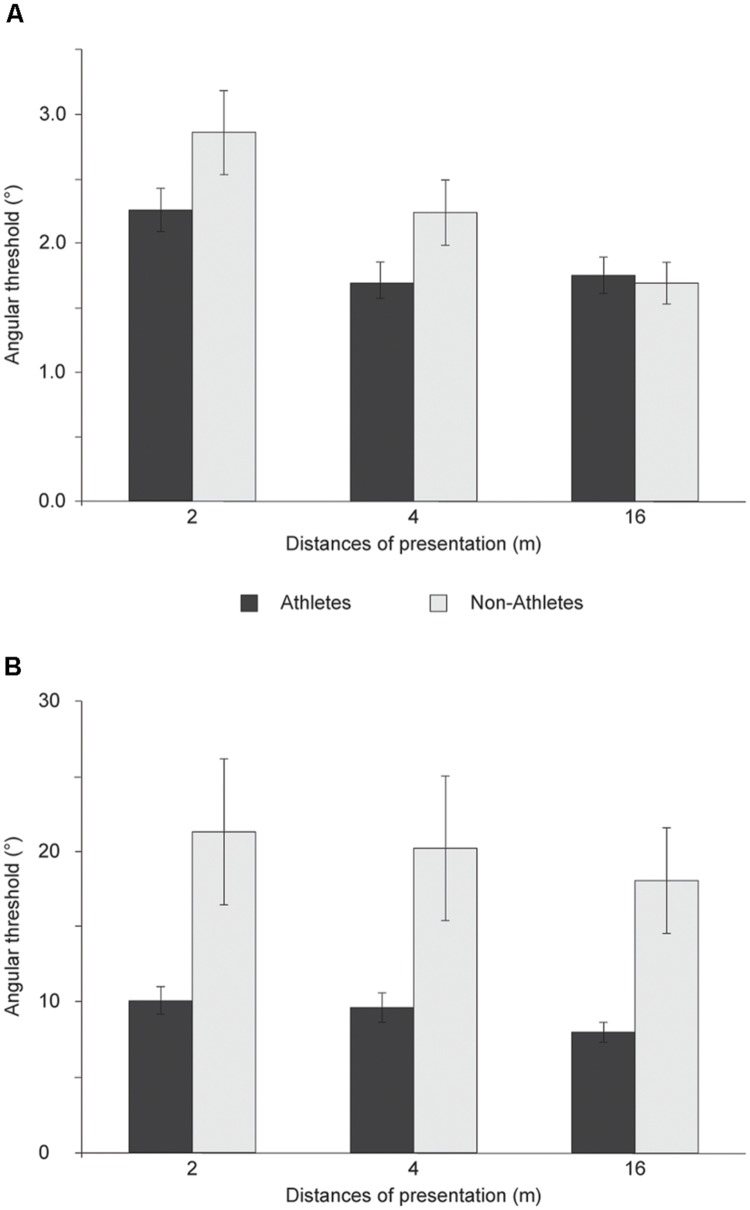
**Athletes and non-athletes’ angular threshold (mean) extrapolated from response accuracy to the direction of a: **(A)** point-light walker and **(B)** point-light soccer kick for multiple distance of presentation**.

The analysis of angular thresholds revealed a significant interaction between distances × groups which reflected a difference in BMP between the groups for the different distances of presentation [*F*(2,114) = 4.586, *p* = 0.012, η^2^ = 0.074; **Figure 2A**]. Independent *t*-tests showed a significant difference in BMP between groups at 4 m of distance [*t*(57) = -2.051, *p* = 0.045], a nearly significant difference at 2 m of distance [*t*(57) = -1.934, *p* = 0.058], and no significant difference at 16 m [*t*(57) = 0.276, *p* = 0.783]. The slope analysis demonstrated a significant difference in BMP between groups at 4 m of distance (*U* = 257.0, *p* = 0.046) but no significant difference at 2 m (*U* = 300.0, *p* = 0.194) and 16 m (*U* = 307.0, *p* = 0.236).

#### Soccer

The analysis of angular thresholds demonstrated a significant difference in BMP between groups at 4 m (*U* = 211.0, *p* = 0.048) and 16 m of distance (*U* = 176.0, *p* = 0.009) but not at 2 m (*U* = 231.5, *p* = 0.108; **Figure 2B**). Whereas there was no significant interaction between distances × groups [*F*(2,108) = 1.772, *p* = 0.175, η^2^ = 0.032], there was a significant difference in the slopes between groups at 4 m [*t*(54) = 2.085, *p* = 0.042] and 16 m of distance [*t*(54) = 2.847, *p* = 0.006] but not at 2 m [*t*(54) = 1.709, *p* = 0.093].

#### Walker vs. Soccer Kick

**FIGURE 3:**
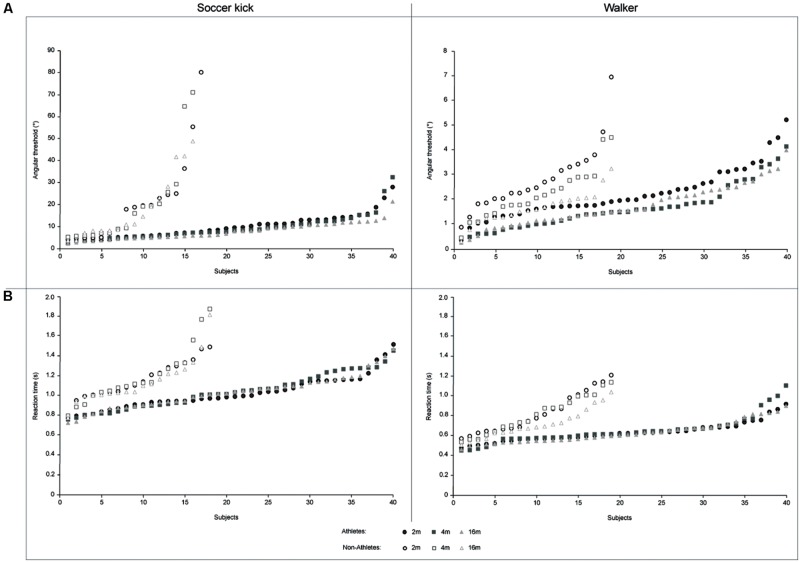
**Representation of response accuracy and reaction time as a function of each subject. (A)** Athletes and non-athletes’ individual angular threshold as a function of subject for the soccer kick (left) and the walker (right) tasks; **(B)** Athletes and non-athletes’ individual reaction time as a function of subject for the soccer kick (left) and the walker (right) tasks.

The analysis of angular thresholds revealed a strong significant effect of the task [*F*(1,55) = 50.414, *p* < 0.001, η^2^ = 0.478] which highlighted the complexity of the soccer kick task compared to the walker task. There was also a general significant effect of distances [*F*(2,110) = 4.980, *p* = 0,009, η^2^ = 0.083]. The analysis of slopes demonstrated the same effects for task [*F*(1,55) = 162.613, *p* < 0.001, η^2^ = 0.747] and distances [*F*(2,110) = 9.209, *p* < 0.001, η^2^ = 0.143]. Participants’ individual performance for the two tasks has been plotted (**Figure 3A**).

#### Inversion Effect

A strong significant effect of orientation (upright, inverted) was revealed throughout the walker task [*F*(1,57) = 187.428, *p* < 0.001, η^2^ = 0.767]. However, there was no significant difference between groups in the inverted condition at 2 m [*t*(57) = 1.457, *p* = 0.151], 4 m [*t*(57) = 1.582, *p* = 0.119], and 16 m [*t*(57) = 0.900, *p* = 0.372]. The same inversion effect was demonstrated in the soccer kick task at 2 m (*Z* = -6.028, *p* < 0.001), 4 m (*Z* = -5.646, *p* < 0.001), and 16 m (*Z* = -5.878, *p* < 0.001) of distances. Contrary to the walker task, there was a difference between groups in the response accuracy at 2 m (*U* = 233.0, *p* = 0.017), 4 m (*U* = 225.0, *p* = 0.012), and 16 m (*U* = 247.5, *p* = 0.031) in inverted condition.

### Reaction Time (**Table 3**)

**Table 3:** Mean (±SEM) reaction time between groups in the two tasks.

Reaction time (s)							
Point-light		Walker			Soccer kick		
	Group	Athletes	Non-athletes	*p*-value	Athletes	Non-athletes	*p*-value
Distance and orientation	2 m Upright	0.63 ± 0.02	0.83 ± 0.05	^∗^0.000	1.02 ± 0.03	1.14 ± 0.05	^∗^0.019
	2 m Inverted	0.73 ± 0.03	0.91 ± 0.07	^∗^0.030	1.12 ± 0.06	1.31 ± 0.07	^∗^0.010
	4 m Upright	0.65 ± 0.02	0.81 ± 0.04	^∗^0.004	1.04 ± 0.03	1.18 ± 0.07	^∗^0.025
	4 m Inverted	0.72 ± 0.03	0.91 ± 0.08	^∗^0.016	1.05 ± 0.03	1.14 ± 0.04	0.104
	16 m Upright	0.63 ± 0.02	0.72 ± 0.04	^∗^0.026	1.03 ± 0.03	1.11 ± 0.06	0.138
	16 m Inverted	0.74 ± 0.03	0.90 ± 0.08	^∗^0.015	1.09 ± 0.03	1.19 ± 0.04	^∗^0.033

*p < 0.05.

#### Walker

The analysis demonstrated a significant difference between groups at 2 m (*U* = 141.0, *p* < 0.001), 4 m (*U* = 200.0, *p* = 0.004), and 16 m (*U* = 243.0, *p* = 0.026) of distance.

#### Soccer

Whereas there was no significant interaction between distances × groups [*F*(2,114) = 0.686, *p* = 0.506, η^2^ = 0.012], there was a significant difference between groups at 2 m [*t*(57) = -2.416, *p* = 0.019] and 4 m [*t*(57) = -2.304, *p* = 0.025] but not at 16 m [*t*(57) = -1.505, *p* = 0.138] of distance.

#### Walker vs. Soccer Kick

There was a strong significant effect of the task [*F*(1,58) = 230.431, *p* < 0.001, η^2^ = 0.799] which highlighted the complexity of the soccer kick task compared to the walker task. There was also a general significant effect of distances [*F*(2,116) = 3.309, *p* = 0,040, η^2^ = 0.054]. Participants’ individual performance for the two tasks has been plotted (**Figure 3B**).

#### Inversion Effect

A significant inversion effect was demonstrated in the walker task at 2 m (Z = -2.914, *p* = 0.004), 4 m (*Z* = -2.657, *p* = 0.008), and 16 m (*Z* = -4.499, *p* < 0.001) of distances. Moreover, there was a significant difference between groups in the inverted condition at 2 m (*U* = 246.0, *p* = 0.030), 4 m (*U* = 232.0, *p* = 0.016), and 16 m (*U* = 230.0, *p* = 0.015). A significant effect of orientation was also revealed throughout the soccer task [*F*(1,57) = 4.736, *p* = 0.034, η^2^ = 0.770]. In addition, there was a significant difference between groups in the inverted condition at 2 m [*t*(57) = -2.680, *p* = 0.010] and 16 m [*t*(57) = -2.179, *p* = 0.033] but not at 4 m [*t*(57) = -1.654, *p* = 0.104].

## Discussion

For the first time, the present study explored the level of expertise of young adult soccer players and non-athlete adults in both sport-specific and non-sport specific BMP contexts by using a 3D point-light walker and soccer kick. As expected, athletes performed better than non-athletes throughout the domain-specific task (soccer kick); but more interestingly, they also showed a greater performance than non-athletes toward a more fundamental and common task such as the facing discrimination of the direction of a human walker. This was particularly obvious when the participant was tested at the critical distance for collision avoidance (4 m). Athletes also demonstrated higher performance than non-athletes in inverted conditions. We did not find a speed-accuracy tradeoff between the two groups, however, soccer players produced more correct answers within shorter reaction times which highlighted their superior performance. According to these results, athletes appear to demonstrate a general, non-sport specific, perceptual-cognitive advantage that benefits to the recognition of human body kinematics in everyday life.

### Sport Specific Expertise

The results obtained from the point-light soccer kick task demonstrated that experts were more accurate (4 m, 16 m) and faster (2 m, 4 m) than non-athletes in predicting the direction of the soccer kick. This result confirms previous literature, which showed evidence of athlete’s perceptual-cognitive expertise in sport-specific environment. Examples using BMP have shown that racquet sport experts showed better prediction accuracy on stroke direction than non-experts (Abernethy et al., 2001; Ward et al., 2002; Abernethy and Zawi, 2007) and that professional basketball players were faster and more accurate than novices in recognizing basketball dribbles (Hohmann et al., 2011). Results from those studies suggested that the perceptual-cognitive advantage is directly related to the athletes’ superior pick-up of essential kinematic information. In our soccer kick experiment, the main difficulty was to be able to predict the direction of the kick from the kinematic of the body motion alone because the ball was masked. In a similar experiment using the French bowling game of ‘pétanque,’ Munzert et al. (2010) tested participant’s prediction of the length of ones throw (Munzert et al., 2010). The movement was displayed explicitly; however, the outcome was masked and left for the participant to anticipate. The authors argued that the prediction of the outcome had to be extrapolated, because no direct information was available on the ball trajectory. Participants clearly rely on body movement features when predicting object properties that occur subsequent to the movement. In soccer, but also in racquet ball games, there is insufficient time to fully analyze the trajectory of the ball before making a preparatory response for a return shot. Consequently, perceptual expertise is reliant on the anticipation based on an opponents’ bodily movements and thus experts are able to identify these important cues prematurely (Wright et al., 2011). One potential mechanism given for the process of anticipation is that the observer simulates the observed actions by using a predictive forward simulation which estimates the sensory effects of a movement. The prediction is suggested to be based on one’s movement experience and/or the observer’s own motor representations (see Bischoff et al., 2015). In a recent fMRI study, Bischoff and colleagues identified the core components of the anticipation network throughout an anticipatory task of boules’ throw (Bischoff et al., 2015).

Moreover, when taking a closer look at the observer’s reaction times in discriminating the kick direction, the results indicate that about 1 s is sufficient for the athlete to make a decision whereas a non-athlete needs about 1.15 s. Knowing that the foot-to-ball contact point of the task was settled at 0.6 s and that 0.15 s is usually necessary for the visual system to give a visuo-motor response, the result indicated that athletes were able to answer shortly afterward foot-to-ball contact while non-athletes had to wait until the end of the movement when no other visual information was given about the ball trajectory. This is confirmed by the individual data which showed that the fastest soccer players were able to answer within 0.8 s indicating that the foot-to-ball contact was the key moment for the decision to be elicited. It has already been demonstrated that goalkeepers who are shown films of penalty kicks can anticipate the location of where the ball will arrive at levels above chance before foot-to-ball contact (Savelsbergh et al., 2002, 2005).

In addition, the result of the soccer experiment also supports that BMP is learned through experience. From BMP studies, innate predisposition of the visual system for BMP (with orientation specificity) have been found in naïve newborn babies (Simion et al., 2008). But it is rather suggested that BM sensitivity depends on prior exposure or familiarity with a stimulus. In fact, a number of studies have shown that visual and motor expertise enhanced BMP (Casile and Giese, 2006; Calvo-Merino et al., 2010). Furthermore, it was also demonstrated that an individual’s own motor representations are activated more effectively by more familiar movements (Bischoff et al., 2012). Our study supports that an individuals’ involvement in soccer increased BMP for soccer-specific kinematic scenes.

### General Perceptual-Cognitive Expertise

In addition, a more striking result revealed that soccer players were also superior to non-athletes in accurately (~2 m, 4 m) and rapidly (2 m, 4 m, 16 m) discriminating motion direction in the front-facing BMP point-light walker task, in particular for distances which are critical for collision avoidance (Legault et al., 2012). Compared to non-athletes, their accuracy was also more consistent across the different distances of presentation which manifests a better capability to perceive body kinematics whether it is presented at close or far distances. The human walker is a general and common kinematics that both athletes and non-athletes are exposed to in their daily lives. They can be both considered as expert perceivers of human walkers. However, the result of our experiment reveals that athlete’s perceptual-cognitive expertise advantage generalizes to the perception of ‘non-sport specific’ body kinematics.

A number of studies have observed that sport expertise is linked with fundamental cognitive and perceptual functions outside the sport-specific domain (Nougier et al., 1991). It is well accepted that physical activity enhances brain plasticity and improves cognitive and executive functions (for recent reviews see Erickson et al., 2013; Vivar et al., 2013). For example, a significant correlation has been demonstrated between the results from the executive functions tests (neuropsychological assessment tool) vs. the number of goals and assists the players had scored two seasons later (Vestberg et al., 2012). The authors suggested that results in cognitive function tests predict the success of top-soccer players. Furthermore, higher order cognitive function have been suggested to be relevant in identifying new talent and development in youth soccer players (Verburgh et al., 2014). Other studies have revealed that athletes outperformed non-athletes in socially realistic multitasking crowd scenes involving pedestrians crossing streets (Chaddock et al., 2011). It was suggested that cognitive skills trained in sport may have transferred positively on to everyday multitasking abilities. Another example showed athletes’ superiority in learning complex and neutral dynamic visual scenes using the 3D-MOT task (Faubert, 2013). The results showed a clear distinction between the level of athletic performance and corresponding fundamental mental capacities for learning an abstract and demanding dynamic scene task void of sports context. The two last-cited paradigms intended to activate and measure the higher-level cognitive abilities subserved by the central nervous system which may play a more general, rather than specific role in sport expertise (Voss et al., 2010). According to our results on BMP with the point-light walker scenario, we can hypothesize that the involvement in a sport activity, e.g., soccer, would transfer advantages to other tasks such as BMP, thus improving BMP sensitivity.

This result is supported by imagery studies showing that BMP induced a selective activation of the brain, especially in the superior temporal sulcus (STS; Oram and Perrett, 1994; Vaina et al., 2001; Ptito et al., 2003). This region is part of the action observation network (AON), a system that is involved in action perception. The AON is comprized of the inferior frontal gyrus, the dorsal premotor cortex, the inferior parietal cortex, the superior parietal cortex, the inferior parietal sulcus, the primary somatosensory cortex, the posterior medial temporal gyrus, the fusiform face/body area, the visual area V5 and more recently the cerebellum (Caspers et al., 2010; Balser et al., 2014a,b). Studies on expertise investigated how the acquisition of a skilled action (e.g., sport moves) affects AON activity while observing the same movement. Those studies revealed a stronger activation for experts in comparison to novices not limited to the AON but recruiting also other brain regions (Turella et al., 2013; Balser et al., 2014a,b). For instance, Balser et al. (2014b) demonstrated an increased activation in areas that subserve the AON following anticipation of tennis strokes in experts and novices. Interestingly, they showed a stronger activation in experts compared to novices demonstrating that neural processing of anticipation depends on the expertise level. At the same time, the expert group outperformed novices on the behavioral level (anticipation task). Moreover, in another study, Balser et al. (2014b) identified the superior parietal cortex as a structure for the processing of domain-specific contextual information (e.g., a domain-specific motor repertoire built up with experience) and the cerebellum as a structure for the storage of internal forward models that allow a rapid prediction of the action outcomes (Balser et al., 2014a). Recently, an imagery study in athletes and non-athletes revealed an increase in thickness of the STS, which was shown to be correlated to the level of sports training (Wei et al., 2011). All of the evidence suggests that the athletes are much better at perceiving movements performed by others, even when insufficient perceptive information is provided.

### Inversion Effect

Regarding the inversion effect, our results are consistent with previous studies, showing a better performance (accuracy and reaction time) for the upright condition than for the inverted condition (Dittrich, 1993; Pavlova and Sokolov, 2000; Legault et al., 2012). This inversion effect is generally attributed to global representations learned in a particular orientation. To exemplify this phenomenon called ‘face inversion effect,’ upright faces are more accurately and rapidly recognized when presented in their canonical orientation rather than presented upside-down (Yin, 1969).

Furthermore, the performance during inverted conditions was slightly better in athletes than non-athletes for point-light walker (reaction time) and soccer kick (accuracy and reaction time) tasks. It has recently been revealed that soccer players exhibit enhanced visual-spatial abilities such as faster reaction time to process rotated embodied stimuli compared to non-athletes (Jansen et al., 2012). Those previous results are further supported by our own data.

### Exploring Expertise using BMP

In the present study, we also demonstrated that the levels of performance (accuracy and reaction time) to discriminate a point-light walker compared to a point-light soccer kick task were strongly different. Mainly to avoid ceiling effect caused by expertise, we decided to use point-light motions requiring different types of expertise ranging from a complex sport-specific environment to a motion we perceive in our every-day life. Earlier, Dittrich (1993) had shown that locomotory actions such as walking were recognized more accurately and faster than social and instrumental actions such as dribbling a basketball or boxing (Dittrich, 1993). The results of the present study support Dittrich’s findings. It is known that observers rely mostly on the feet to discriminate left from right-facing point-light walkers (Troje and Westhoff, 2006; Chang and Troje, 2009; Saunders et al., 2010). Other evidence have shown that experienced and naive observers can also use information about the body structure (global analysis of the entire human body) to judge the walking direction during a facing task from time-scrambled sequences (Lange and Lappe, 2007) or even from static (‘snapshots’) stimuli (Reid et al., 2009). An interesting question to address in the future would be to assess whether athletes are superior to non-athletes in the total absence of kinematics while using the same static stimuli as in the Reid et al. (2009) study. On the other hand, discriminating the direction of a soccer kick may require different sources of information. Evidence from film analysis and eye tracker studies suggested that experts relied on local information to anticipate the penalty kick from a striker. Generally, the local motion was defined by the orientation of the non-kicking foot (Franks and Hanvey, 1997; Savelsbergh et al., 2002, 2005). However, within the same studies, fixation on local information represented only a small portion of total fixation duration and motion information was picked up by the periphery in areas off of the foot and ball region (Savelsbergh et al., 2002, 2005). This evidence suggests that, the anticipation of a kick does not simply rely on local information illustrated by the angle of the non-kicking foot. In fact, the action of kicking a ball while maintaining stability is a complex kinematic that involves the participation of the arms, legs, torso, and head; therefore, motion components may be distributed across the body rather than localized to a particular limb segment as reflected while walking. Diaz et al. (2012) identified a list of sources of information used by subjects to judge the direction of a kick: the yaw angle of the hips, contact yaw, and two sources of distributed information (Diaz et al., 2012). They suggested that kick direction was perceived on the basis of distributed information, possibly in conjunction with a reliable source of local information (e.g., contact yaw). Distributed (soccer) vs. local (walker) information could help explain the differences observed between our two BMP tasks.

Furthermore, we used varying angles of presentation throughout BMP paradigms to avoid ceiling effect. As shown earlier by Saunders et al. (2010), varying viewing angles produces a change in accuracy; such that near frontal views (e.g., close to 0°) induced a lower level of response accuracy than more side views (e.g., 15°; Saunders et al., 2010). Taken together, the results confirmed that varying the viewing angle during a facing task and the nature of the task increases BMP difficulty and therefore is an appropriate technique to explore levels of expertise in human.

## Conclusion

Throughout this study, we were able to highlight the sport specific perceptual-cognitive expertise of soccer players using BMP. Furthermore, an interesting finding revealed a general perceptual-cognitive advantage in athletes, or enhancement of BMP sensitivity, for perceiving a fundamental kinematic of action (walker) compared to non-athletes. This result is in keeping with recent evidence of athletes’ perceptual skill transfer to everyday activities involving perceptual-cognitive abilities. It also supports previous findings from imagery studies showing enhanced cognitive activity in specific brain regions underlying action-perception processes. As expected, we observed an inversion effect but we also demonstrated that athletes were slightly better than non-athletes for the inverted condition. On the whole, the BMP paradigm is an appropriate measure for demonstrating expertise. It lends itself to manipulation of multiple parameters in order to assess specific properties of expert perceptual-cognitive skills.

## Conflict of Interest Statement

The authors declare that the research was conducted in the absence of any commercial or financial relationships that could be construed as a potential conflict of interest.
